# Catalytic pyrolysis of waste rice husk over mesoporous materials

**DOI:** 10.1186/1556-276X-7-18

**Published:** 2012-01-05

**Authors:** Mi-Jin Jeon, Seung-Soo Kim, Jong-Ki Jeon, Sung Hoon Park, Ji Man Kim, Jung Min Sohn, See-Hoon Lee, Young-Kwon Park

**Affiliations:** 1 Graduate School of Energy and Environmental System Engineering, University of Seoul, Seoul, 130-743, Korea; 2 Department of Chemical Engineering, Kangwon National University, Samcheok, 245-711, Korea; 3 Department of Chemical Engineering, Kongju National University, Cheonan, 330-717, Korea; 4 Department of Environmental Engineering, Sunchon National University, Suncheon, 540-742, Korea; 5 Department of Chemistry, BK21 School of Chemical Materials Science and Department of Energy Science, Sungkyunkwan University, Suwon, 440-746, Korea; 6 Department of Mineral Resources and Energy Engineering, Chonbuk National University, Jeonju, 561-756, Korea; 7 School of Environmental Engineering, University of Seoul, Seoul, 130-743, Korea

**Keywords:** Py-GC/MS, rice husk, Meso-MFI, Pt-Meso-MFI

## Abstract

Catalytic fast pyrolysis of waste rice husk was carried out using pyrolysis-gas chromatography/mass spectrometry [Py-GC/MS]. Meso-MFI zeolite [Meso-MFI] was used as the catalyst. In addition, a 0.5-wt.% platinum [Pt] was ion-exchanged into Meso-MFI to examine the effect of Pt addition. Using a catalytic upgrading method, the activities of the catalysts were evaluated in terms of product composition and deoxygenation. The structure and acid site characteristics of the catalysts were analyzed by Brunauer-Emmett-Teller surface area measurement and NH_3 _temperature-programmed desorption analysis. Catalytic upgrading reduced the amount of oxygenates in the product vapor due to the cracking reaction of the catalysts. Levoglucosan, a polymeric oxygenate species, was completely decomposed without being detected. While the amount of heavy phenols was reduced by catalytic upgrading, the amount of light phenols was increased because of the catalytic cracking of heavy phenols into light phenols and aromatics. The amount of aromatics increased remarkably as a result of catalytic upgrading, which is attributed to the strong Brönsted acid sites and the shape selectivity of the Meso-MFI catalyst. The addition of Pt made the Meso-MFI catalyst even more active in deoxygenation and in the production of aromatics.

## Introduction

Due to the increasing cost of crude oil and the environmental problems stemming from the overuse of fossil fuels, it has become increasingly important to develop alternative forms of renewable energy. Among these, biomass is regarded as a promising renewable energy source, and research on its application is being conducted extensively all over the world. In principle, compared to conventional fossil fuels, biomass does not cause a net carbon dioxide increase in the atmosphere, and it contains lower amounts of sulfur and nitrogen. Therefore, the use of biomass has benefits in terms of mitigating climate change as well as addressing the problem of air pollution [1-3].

Fast pyrolysis, in which biomass is decomposed thermochemically, is attracting a large amount of attention as a practical way to produce alternative liquid fuel that can replace fossil fuels. The liquid product of fast pyrolysis, known as bio-oil, can be used not only as fuel, but also as a raw material in the production of high-value-added chemicals. However, bio-oil has several problems that hinder its direct application to conventional combustion engines. These drawbacks include poor miscibility with petroleum oils, high oxygenate content, corrosivity against metals, and thermochemical instability [4,5]. Various methods to upgrade bio-oil have been investigated. Catalytic cracking converts oxygenates such as aldehydes and ketones into low-molecular-mass species with low oxygen content through a catalytic reaction. During the process, the oxygen atoms contained in the bio-oil are converted into H_2_O, CO, and CO_2 _and removed to improve the bio-oil's quality [6]. Microporous zeolites such as ZSM-5, Y, and Beta have been widely applied for the catalytic upgrading of bio-oil. However, the pore size of these zeolites is so small (< 1 nm) that the pores can be blocked easily and it is difficult for large-sized bio-oil molecules to diffuse into the pores. Meso-MFI zeolite [Meso-MFI] is known to be a powerful catalyst that has strong acid sites like microporous zeolites as well as a large pore size [7].

Recently, various agricultural byproducts and wastes, including straw, olive seed, and nut shell, have been applied to the research of fast pyrolysis [8]. Rice husk is an abundant biomass resource that is produced in the agricultural society of Korea. Previously, waste rice husk was usually composted or incinerated. These conventional treatment methods, however, are not adequate for treating organic waste because they can cause nitrogen deficiency in the composting and smoke emissions. Fast pyrolysis of rice husk can be an alternative way to treat this material because it not only treats organic waste more efficiently, but it also produces bioenergy [9].

In this study, Meso-MFI, which has adequate characteristics for catalytic upgrading of bio-oil, was used for the first time for the catalytic pyrolysis of rice husk. Pyrolysis-gas chromatography/mass spectrometry [Py-GC/MS] was used to analyze the pyrolysis products directly and to examine the effect of the catalyst.

## Experimental details

### Rice husk

Rice husk was supplied from a rice mill located in Jeonnam, Korea. It was dried in an oven for 24 h at 110°C to minimize the effect of moisture. The sample particles were 8 to 10 mm long, 2.0 to 2.5 mm wide, and 0.1 to 0.15 mm thick. For each experiment, 1 mg of the sample was used.

Ultimate analysis was performed using a TruSpec elemental analyzer (LECO Co., St. Joseph, MI, USA) and an SC-432DR sulfur analyzer (LECO Co., St. Joseph, MI, USA) to quantify C, H, O, N, and S. Proximate analysis was carried out using a thermogravimetric analyzer (Pyris 1 TGA, PerkinElmer, Waltham, MA, USA).

### Catalyst synthesis

Meso-MFI with a Si-to-Al ratio of 20 was synthesized by following the procedure described in the literature [7]. A 0.5-wt.% platinum [Pt] was ion-exchanged into Meso-MFI using Pt(NH_3_)_4_(NO_3_)_2_. Calcination for 3 h at 500°C under O_2 _atmosphere and H_2 _reduction was applied to the Pt-Meso-MFI before use [10].

### Characterization of catalysts

To examine the specific surface area, pore volume, and pore size distribution of the catalysts used in this study, nitrogen adsorption-desorption was measured at 77 K (BELSORP-MINI, BEL Japan, Inc., Osaka, Japan) for the catalysts pretreated at 200°C under vacuum condition. From the obtained adsorption-desorption isotherms, the specific surface area was determined using the Brunauer-Emmett-Teller [BET] method.

To investigate the acidic properties of the catalysts, NH_3 _temperature-programmed desorption [TPD] analysis was carried out using a TPD/TPR 2900 analyzer (Micromeritics Instrument Co., Norcross, GA, USA). Before each analysis, the sample was pretreated with He gas at 250°C and was then cooled down to 100°C. While heating the sample from 100 to 400°C at a rate of 10°C/min with a N_2 _flow rate of 50 mL/min, the amount of NH_3 _desorbed from the catalyst was measured using a thermal conductivity detector.

### Pyrolysis-gas chromatography/mass spectrometry

Py-GC/MS experiments were performed using a vertical furnace type pyrolyzer (Py-2020D, Frontier-Lab Ltd., Fukushima, Japan). About 1 mg of the rice husk sample was put on the metal sample cup floor. An intermediate layer made of quartz wool was installed above the sample to prevent contact between the catalyst and the sample. About 1 mg of the catalyst was located above the quartz wool layer. Therefore, catalytic upgrading was supposed to take place when the vapor-phase pyrolysis products passed through the catalyst layer. The metal sample cup containing the catalyst and the sample was inserted into the preheated pyrolyzer through which helium carrier gas flowed.

Vapor species produced from pyrolysis reactions for 3 min at 500°C were analyzed directly using a GC (HP 6890N Gas Chromatography)/MS (HP 5973 inert Mass Spectral Detector, Agilent Technologies Inc., Santa Clara, CA, USA) connected to the pyrolyzer. An HP-5 MS (30 m × 0.25 mm × 0.25 μm) capillary column was used for the analysis. Carrier gas was supplied with a split ratio of 50:1, and the GC/MS interface temperature was controlled at 300°C. The GC oven temperature was programmed to rise from 40 to 300°C at a rate of 5°C/min so that the analysis was conducted for 66 min, including 4 min maintained at 40°C before heating and 10 min maintained at 300°C after heating. Each peak appearing in the obtained mass spectra was interpreted using the NIST05 library.

## Results and discussion

### Sample analysis

The physicochemical properties of waste rice husk are displayed in Table 1. A rice husk consists mainly of carbon and oxygen, and the oxygen content was relatively high (38.1%), which may be attributed to oxygen-containing functional groups such as hydroxyl and carbonyl groups that are abundant in rice husks. Combustibles accounted for 78.8%. Compared to general wood biomass containing less than 1 wt.% ash, the rice husk was shown to consist of more than 10 wt.% of ash, which can be a drawback in the production of bio-oil. Sulfur was not detected, implying that the produced bio-oil will be a clean fuel.

**Table 1:** Physicochemical characteristics of rice husk

**Elemental analysis**^**a**^		Proximate analysis	
Component	Content (wt.%)	Component	Content (wt.%)
C	53.5	Moisture	9.3
H	7.0		
N	1.4	Combustibles	78.8
O^b^	38.1		
S	-	Ash	11.9

^a^On dry and ash free basis; ^b^calculated by difference.

### Catalyst characterization

Table 2 displays the physical properties of the catalysts used in this study. The specific surface area of Meso-MFI was larger than that of the commercial MFI catalyst (411 m^2^/g), and the pore size was about 4.1 nm, which is large enough to allow bio-oil molecules to diffuse into the pores. When Pt was added, the specific surface area decreased slightly due to the blocking of the pores by the Pt.

**Table 2:** Physical properties of the catalysts

	*S*_BET _(m^2^/g)	*V*_tot _(cm^3^/g)	Average pore size (nm)	Si/Al ratio
Meso-MFI	567	0.7	4.1	15
Pt-Meso-MFI	472	0.6	4.1	15

**Figure 1:**
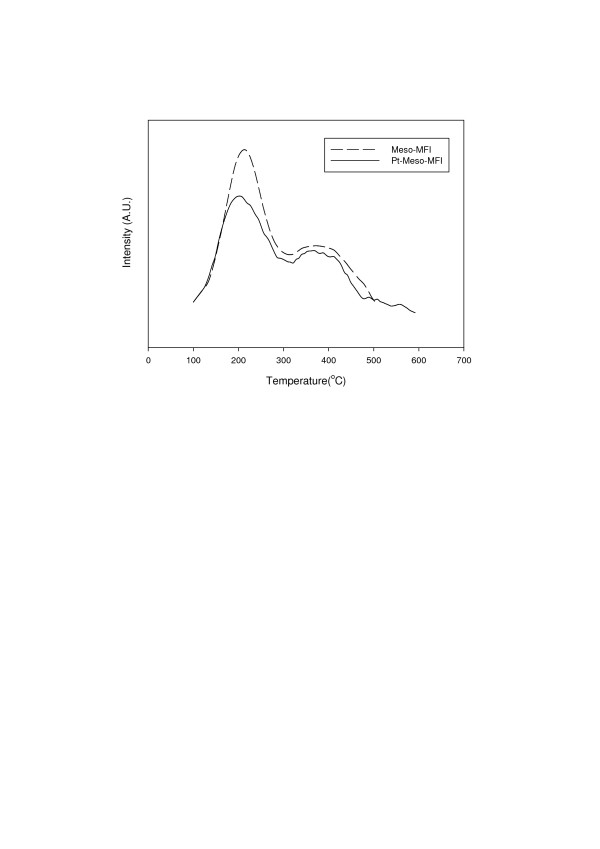
**NH_3_-TPD curves of catalysts**.

Figure 1 shows the result of temperature programmed desorption of ammonia [NH_3_-TPD] analysis performed to examine the acid properties of the catalysts. Meso-MFI exhibited two peaks: a low-temperature peak at about 230°C and a high-temperature peak at about 400°C. The low-temperature peak represents weak acid sites, while the high-temperature peak represents the strong Brönsted acid sites [7]. As with a typical microporous MFI catalyst, Meso-MFI has both weak acid sites and strong acid sites. When Pt was added, the amount of acid sites decreased because Pt replaced some of them, but strong acid sites still existed.

### Catalytic upgrading using Py-GC/MS

To analyze the distribution of pyrolysis products easily and accurately, the Py-GC/MS method was used. Fast pyrolysis was carried out at 500°C, which has been suggested to be the optimal temperature for catalytic pyrolysis of biomass [7]. It is well known that the product of fast pyrolysis of biomass includes volatile species and nonvolatile oligomers. Py-GC/MS can be used only for the analysis of volatile species. More than 100 peaks appeared in the chromatograms obtained from the fast pyrolysis of rice husk. The number of peaks increased when catalysts were used compared to non-catalytic pyrolysis.

The yields and compositions of the pyrolysis products were changed due to catalytic upgrading. Catalysts can affect the production of volatile liquid products via two pathways during the catalytic pyrolysis process. First, the product species can be cracked into gasses or converted into coke or char, which reduces the volatile liquid species. Second, catalysts decompose nonvolatile oligomers into volatile monomers, increasing the amount of volatile liquid species. The change in the liquid product yield due to catalytic upgrading is determined by a sum of these two opposite effects [11].

**Figure 2:**
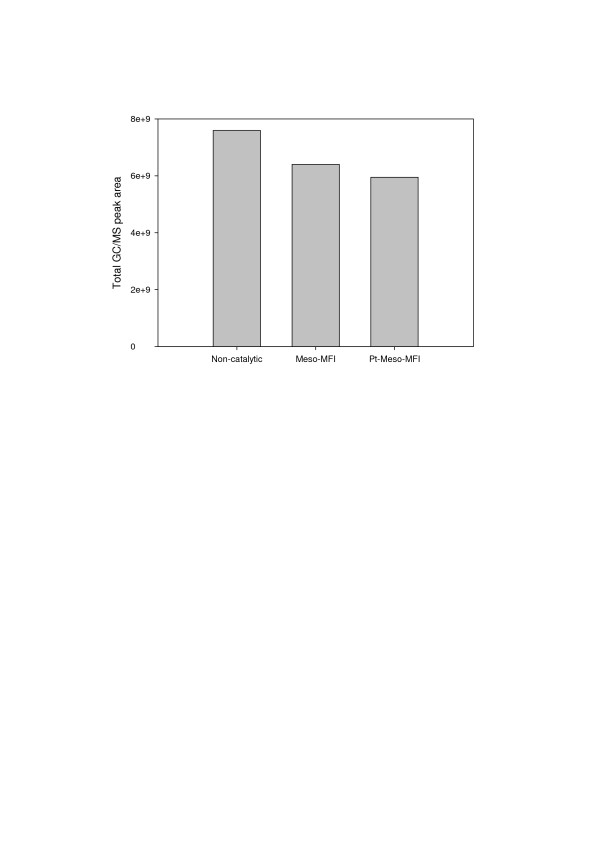
**Total GC/MS peak areas for liquid products from catalytic pyrolysis of rice husk**.

Total GC/MS peak areas of liquid products were plotted in Figure 2 to examine the effects of the catalysts on the yield of volatile liquid products. Because bio-oil contains various species, it is difficult to obtain a quantitative analytic result from GC/MS. Nevertheless, it is possible to compare the product yield by comparing the peak areas because the peak area is proportional to the product yield [12]. The use of catalysts resulted in a decreased total peak area for the liquid product, which may be attributed to cracking of volatile species into CO, C_1_-C_4 _hydrocarbons, and H_2_O. In particular, when Pt was added, the reduction of the total peak area for the liquid product was larger due to a stronger cracking effect. This result indicates that catalytic upgrading reduces the liquid product yield.

**Figure 3:**
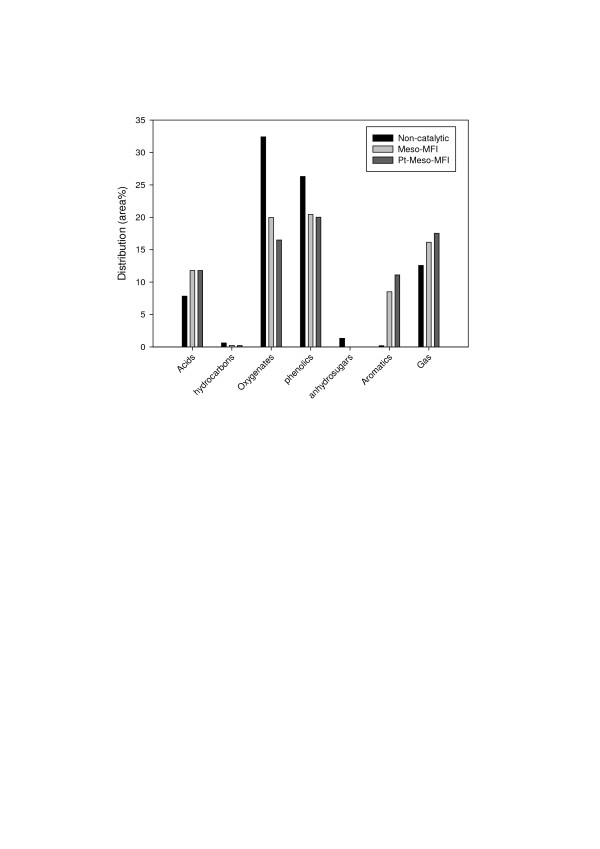
**Product distribution from catalytic pyrolysis of rice husk**.

To investigate the change in the composition of the pyrolysis product due to catalytic upgrading, the pyrolysis products obtained with and without a catalyst were divided into seven groups, i.e., acids, hydrocarbons, oxygenates, phenolics, anhydrosugars, aromatics, and gas. Figure 3 compares the product distributions expressed as area percentage.

As shown in Figure 3, catalytic upgrading reduced the amount of oxygenates due to cracking by the catalysts. In particular, anhydrosugars, which are polymers such as levoglucosan, were cracked into low-molecular-mass species, while aldehydes and ketones were converted into H_2_O by dehydration or into CO and CO_2 _by decarbonylation and decarboxylation. It was observed that catalytic upgrading resulted in an increased gas yield, levoglucosan was decomposed completely, and the amount of carbonyl-containing species was decreased (data not shown). The reduction of total oxygenates due to Meso-MFI and Pt-Meso-MFI was 38% and 49%, respectively. Oxygenates degrade the quality of bio-oil by reducing its heating value and producing air pollutants upon combustion. Therefore, a reduction of oxygenate production by catalytic upgrading may help to produce higher-quality bio-oil. In particular, Pt-Meso-MFI showed a higher deoxygenation effect, which is believed to be due to enhanced cracking, dehydrogenation, hydrogenolysis, and hydrocracking reactions over Pt [13].

**Figure 4:**
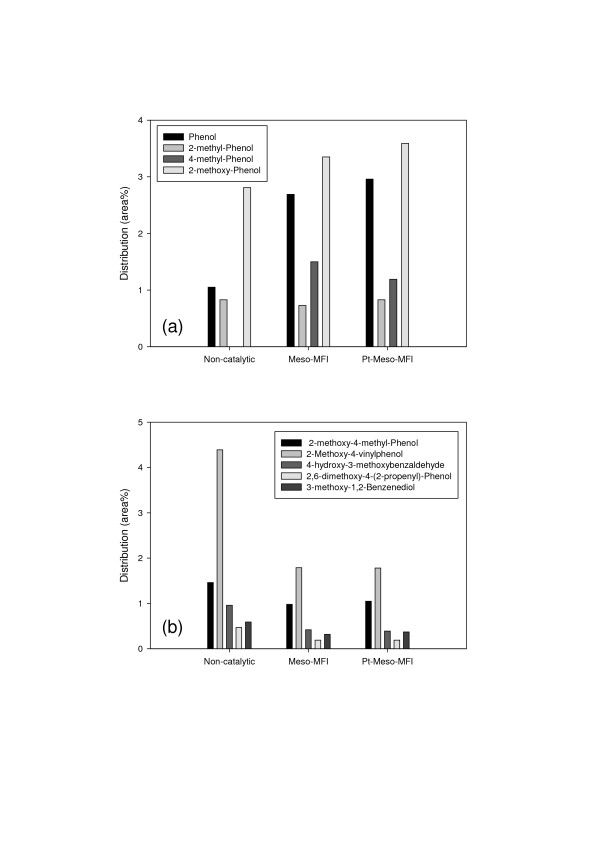
**Effect of catalysts on the phenolic products (a) the light phenol (b) the heavy phenol**.

The total amount of phenolics was reduced due to catalytic upgrading, but the amount of light phenols was increased. This result is attributed to cracking of heavy phenols into light phenols and aromatics due to the outstanding cracking ability of the Meso-MFI catalyst. Figure 4 illustrates this trend clearly. By catalytic upgrading, the amount of phenol and monomeric phenols was increased (Figure 4a), while the amount of heavy phenols was decreased (Figure 4b).

**Figure 5:**
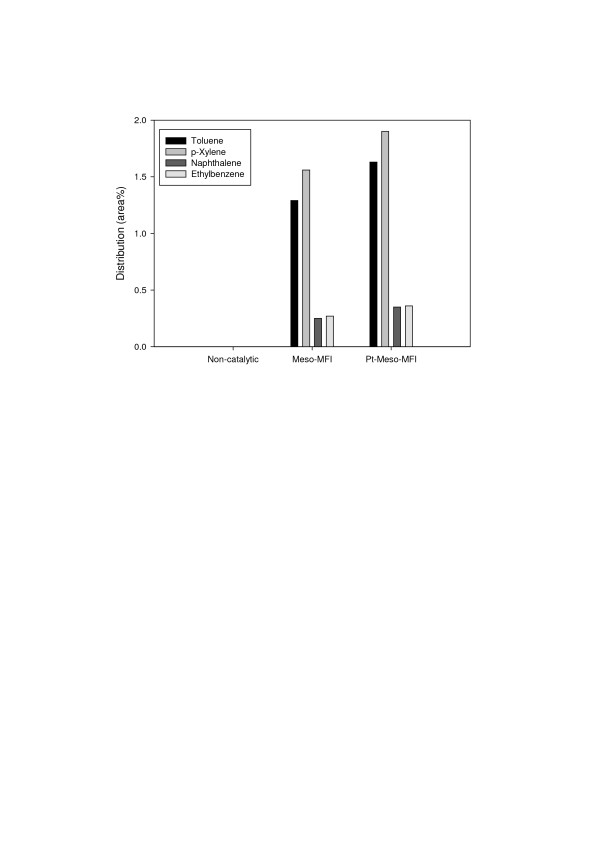
**Effect of catalysts on the aromatic products**.

Aromatics that were not produced by non-catalytic pyrolysis appeared after catalytic upgrading. In particular, the amount of high-value-added mono-aromatics such as toluene and p-xylene increased remarkably (Figure 5). The existence of strong Brönsted acid sites is known to increase the yield of aromatics [7,12]. It has also been reported that the shape selectivity of the MFI structure of MFI catalysts increases the selectivity for aromatics [12]. Therefore, the high selectivity of Meso-MFI for aromatics observed in this study is attributed to its strong Brönsted acid sites and MFI structure. The further increase in the aromatic yield due to the addition of Pt can be explained by the dehydrogenation reactions promoted by Pt. The alkenes produced by dehydrogenation reactions are converted into aromatics on the strong Brönsted acid sites [7].

## Conclusions

In this study, catalytic pyrolysis of waste rice husk was carried out using Py/GC-MS. The catalytic activities of Meso-MFI and Pt-Meso-MFI were evaluated by comparing the product compositions with that of non-catalytic pyrolysis.

The yield of oxygenates that can degrade the product bio-oil was reduced due to catalytic upgrading, by 38% with Meso-MFI and by 49% with Pt-Meso-MFI, demonstrating the outstanding activity of the Pt-Meso-MFI catalyst in deoxygenation. Catalytic upgrading over Meso-MFI increased the amount of light phenols, which is attributed to cracking of heavy phenols into light phenols and aromatics due to the catalytic effect of mesoporous Meso-MFI. In addition, Meso-MFI exhibited a good selectivity for aromatics, which is ascribed to its strong acid sites and shape selectivity due to the MFI structure. Pt-Meso-MFI was even more effective in the upgrading of bio-oil because of the additional catalytic effect of Pt.

## Abbreviations

Meso-MFI: meso-MFI zeolite; NH_3_-TPD: temperature programmed desorption of ammonia; Py-GC/MS: pyrolysis gas chromatography/mass spectrometry.

## Competing interests

The authors declare that they have no competing interests.

## Authors' contributions

MJJ, SSK, JKJ, SHP, JMK, JMS, and SHL participated in some of the studies and in drafting the manuscript. YKP conceived the study, participated in all experiments of this study, and prepared and approved the final manuscript.
